# Venous return and mean systemic filling pressure: physiology and clinical applications

**DOI:** 10.1186/s13054-022-04024-x

**Published:** 2022-05-24

**Authors:** Romain Persichini, Christopher Lai, Jean-Louis Teboul, Imane Adda, Laurent Guérin, Xavier Monnet

**Affiliations:** 1 Service de Réanimation et Soins Continus, Centre Hospitalier de Saintonge, 11 Boulevard Ambroise Paré, 17108 Saintes cedex, France; 2 Université Paris-Saclay, AP-HP, Service de médecine intensive-réanimation, Hôpital Bicêtre, DMU CORREVE, Inserm UMR S_999, FHU SEPSIS, Groupe de Recherche Clinique CARMAS, Le Kremlin-Bicêtre, France

**Keywords:** Fluid therapy, Norepinephrine, Guyton, Heart–lung interactions, Mean systemic filling pressure

## Abstract

Venous return is the flow of blood from the systemic venous network towards the right heart. At steady state, venous return equals cardiac output, as the venous and arterial systems operate in series. However, unlike the arterial one, the venous network is a capacitive system with a high compliance. It includes a part of unstressed blood, which is a reservoir that can be recruited via sympathetic endogenous or exogenous stimulation. Guyton’s model describes the three determinants of venous return: the mean systemic filling pressure, the right atrial pressure and the resistance to venous return. Recently, new methods have been developed to explore such determinants at the bedside. In this narrative review, after a reminder about Guyton’s model and current methods used to investigate it, we emphasize how Guyton’s physiology helps understand the effects on cardiac output of common treatments used in critically ill patients.

**Supplementary Information:**

The online version contains supplementary material available at 10.1186/s13054-022-04024-x.

## Introduction

Cardiovascular physiology is often described by considering the heart and the arterial system as the main actors. This puts at the centre of the paradigm cardiac function and its determinants (preload, afterload and contractility) and the properties of the arterial system (resistance and compliance). However, any attempt to understand shock is pointless if it neglects venous return and its determinants.

In 1884, Baylis and Starling designed the concept of a static vascular pressure at zero flow called the *“mean general blood pressure”* [1]. In 1954, Guyton proposed a model for venous return and defined its determinants, including the “*mean systemic filling pressure*” (Pmsf) [2–4]. He described every haemodynamic condition depending on these determinants [3]. This model has been a matter of controversy [5–9], though it remains the most suitable to describe the pathophysiology of circulatory failure [10].

During recent years, methods enabling the estimation of the determinants of venous return at the bedside have been developed. They have enabled several physiological studies, which were carried out no longer in animals, but in critically ill and post-operative patients. The goal of this narrative review is to show how such studies may change our practice. After having recalled the basic physiology, we will show how these news studies help understand the effects of the most common treatments we use in circulatory failure.

## What generates cardiac output?

One way to figure out cardiovascular system functioning is to think that it is the pressure produced by the left ventricle that generates flow and propels blood from the aorta towards the right atrium [9]. This view is likely inaccurate.

**Fig. 1:**
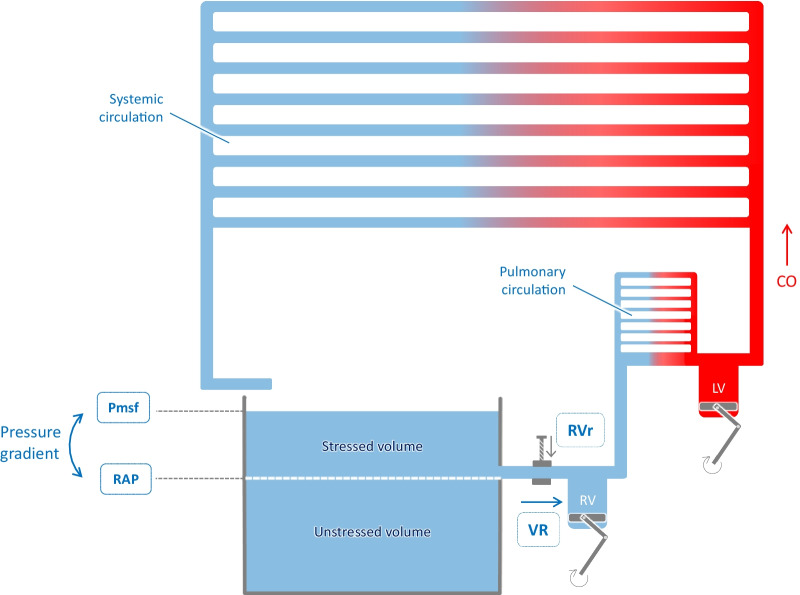
The three determinants of venous return. At equilibrium, cardiac output (CO) and venous return (VR) are similar. Venous return is the inflow of the right heart. Its three determinants are the mean systemic filling pressure (Pmsf), the right atrial pressure (RAP) and the resistance to venous return (RVr) according to the formula: venous return = (Pmsf–RAP)/RVr. LV: left ventricle, RV: right ventricle

According to Guyton’s theory, cardiac output is primarily governed by venous inflow. Systemic blood flow is generated by the pressure difference between the veins and venules on the one hand and the right atrium on the other hand, with the heart emptying the right atrium and keeping the right atrial pressure (RAP) low (Fig. 1). Then, blood flow is created by emptying of the venous system, rather than by ejection into the arterial network.

According to this theory, RAP, i.e. the backward pressure of venous flow, impedes venous return. Guyton postulated that there is a forward pressure that drives flow towards the right atrium, which is Pmsf. This pressure results from the elastic recoil potential stored in the walls of the veins.

In this regard, the role of the heart can be viewed as keeping a significant difference between Pmsf and RAP: the more potent the pump, the lower RAP, the larger the difference between Pmsf and RAP, and the higher the venous return. At each contraction, the heart empties the right ventricle, allowing the elastic recoil pressure in veins and venules to drain blood back to the right atrium. The blood volume that has been removed from the veins and venules to refill the right cavities is then added into the arterial network at the other side [11]. The role of the left heart is to eject the blood towards the high-pressure circuit, to ensure distribution in the resistive system.

## What is the role of the venous system?

According to this model, centred on the importance of venous return, Pmsf, and not the arterial pressure, propels flow towards the right atrium. To explain this, one must consider that veins and venules represent a blood bulk, of large size and of high compliance.

Magder and De Varennes nicely compared the cardiovascular system to a bathtub, filled by a tap and emptied through a hole located in the side of the tub [12] (Fig. 1). The flow out of the tub, likened to venous return, is only determined by the hydrostatic pressure of water above the hole, not by the pressure coming out of the tap, which is likened to cardiac output, because it is low compared to the tub volume. In the “bathtub model”, the venous system plays the role of the tub, and the tap, i.e. cardiac output, only fills the bathtub [12]. The role of the hydrostatic pressure that propels water out of the tub is played by the pressure generated by the elastic recoil of the venous system. The volume that stretches the elastic veins provides the potential energy that generates flow. However, as any elastic structure, e.g. a balloon, veins have a resting volume which does not stress their elastic walls and creates no pressure. This volume which does not contribute to venous flow is called the *unstressed blood volume*. In the Magder and De Varennes bathtub model, it corresponds to the water volume below the hole. Any amount of blood added to the venous reservoir will produce tension in its walls and increase the intravascular pressure. This part of venous blood (water volume above the hole) is the *stressed blood* volume (Fig. 1).

The venous system is large: in an adult human, it contains approximately 70% of the total blood volume, i.e. 3.5 L [13], with about 70% in the splanchnic vascular bed [14, 15]. Moreover, venous compliance is 40 times greater than arterial compliance [16]. In humans under normal conditions, the unstressed blood volume is approximately 70% of the total blood volume [12]. It represents a large reserve of paramount importance that may be recruited by an α-adrenergic-mediated venous constriction [17, 18]. This venoconstriction moves part of the unstressed blood volume to the stressed blood volume [15, 19].

## What are the determinants of venous return?

### The venous return curve

Venous return is the blood flow crossing both venae cavae. According to Poiseuille’s law, it is determined by the resistance to venous return (RVr), and by the pressure gradient between the downstream pressure, i.e. RAP, and the upstream pressure, which is Pmsf (Fig. 1):

$${\text{Venous return }} = \, \left( {{\text{Pmsf}} - {\text{RAP}}} \right)/{\text{RVr}}$$

**Fig. 2:**
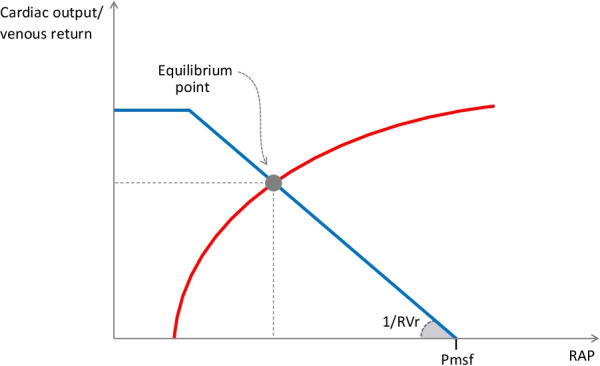
Overlaying of cardiac function curve and venous return curve. The venous return curve (in blue) indicates that venous return decreases when right atrial pressure (RAP) increases. Mean systemic filling pressure (Pmsf) is the pressure at the x-intercept of the venous return curve. Resistance to venous return (RVr) is represented by the inverse of the slope. At the intersection of the venous return curve and the cardiac function curve (in red) stands the point of equilibrium, whose coordinates reflect the haemodynamic conditions

Figure 2 illustrates this physiological concept which was confirmed by Guyton in animal experiments [2–4]. When RAP decreases, the pressure gradient between RAP and Pmsf increases, and venous return increases. When RAP decreases below a critical value, venous return does not increase anymore (Fig. 2). This plateau corresponds to the “vascular waterfall” or “flow limitation” [11] phenomenon, when veins collapse as their intramural pressure becomes lower than the extramural pressure [20, 21].

On this graph, note that Pmsf can be estimated by the x-intercept of the venous return curve, for at zero flow, RAP equals Pmsf. RVr is represented by the inverse of the curve slope (Fig. 2).

### Guyton, Frank and Starling in one figure

The cardiovascular system is a closed loop. At equilibrium, venous return equals cardiac output (Fig. 1). Cardiac output adapts to changes in venous flow through the Starling mechanism [22]. Any increase in ventricular preload increases the degree of interaction between actin and myosin filaments, increasing the intrinsic cardiac contractility and stroke volume. When the myofilaments are stretched at their maximal length, any further increase in cardiac preload does not increase stroke volume anymore: the ventricles become *preload unresponsive*.

As venous return and cardiac output are equal, and as RAP is a marker of cardiac preload, it is possible to superimpose the venous return and the cardiac function curves [3] (Fig. 2). Their intercept defines an operating point at equilibrium. Its coordinates (RAP and venous return/cardiac output) and Pmsf (the x-intercept of the venous return curve) describe the haemodynamic characteristics under the studied condition (Fig. 2). This representation allows the description of all pathological haemodynamic states [22].

## What changes venous return?

Pmsf is determined by the volume of the large compliant venules and small veins. It is close to the mean circulatory filling pressure, which is the pressure to which arterial and central venous pressures converge when there is no flow, as, for instance, during the first seconds after cardiac arrest (later, reflexes and ischaemia-induced vasodilation may change this pressure) [23, 24]. This mean circulatory pressure is generated by the elastic recoil of the whole cardiovascular circuit. In theory, it is different from Pmsf, which is the pressure generated by the elastic recoil of the venules and small veins only. However, distinguishing Pmsf from mean circulatory filling pressure is not clinically important because they are very similar [11, 25], except in some specific circumstances (pulmonary hypertension and very low cardiac output).

The normal value of Pmsf varies among studies and likely among species [2, 26–28]. In normal conditions in humans, Pmsf is 2–10 mmHg [11]. In patients, Pmsf varies depending on clinical circumstances, volume status and vasomotor tone. In cardiac surgery and septic shock patients, values of 15–33 mmHg have been reported [29]. These are higher than in normal conditions, due to treatments such as fluid infusion and vasopressors. The rise in RAP, for example, under positive pressure ventilation [30], also leads to an increase in Pmsf, which maintains a sufficient pressure gradient of venous return. During cardiac arrest, lower values are estimated, which is explained by a different haemodynamic state [29, 31].

RAP is mainly influenced by the efficiency of the cardiac pump: the more efficient, the lower the RAP. Also, RAP is affected by changes in pleural pressure during ventilation, which contributes to the respiratory variation of stroke volume. Note that the value of RAP which opposes venous return is the intramural pressure of the right atrium, not the transmural pressure.

RVr is mainly influenced by vein diameter. It is under the control of the sympathetic system. RVr may also increase in the case of high extramural pressure. Two factors of equal importance can modify Pmsf. The first is the volume of blood in the venous reservoir, which is increased by fluid administration. The second is the capacitance of the venous system, which is under the control of adrenergic tone.

The concept of resistance to venous return is perhaps more complex, even if the description we gave through Poiseuille’s law helps to understand the clinical applications of physiology. Indeed, Guyton observed that changes in resistance have a major effect on venous return when it occurs near the right atrium, but a lesser effect when the changes are made further away. He suggested defining “peripheral resistance to venous return” as the sum of venous and arterial resistance [3].

## How to measure the determinants of venous return?

Years after the animal studies of Guyton [3, 4], methods have been developed to approach the determinants of venous return at the bedside. They are of no interest for routine management, but are now used in physiological studies. These different methods are described in more detail in the supplementary material (Additional file 1).

### Cardiac arrest

The first measurements of Pmsf in humans were made during electrophysiologic investigations. The pressure to which arterial pressure and central venous pressure (CVP) converge after ventricular fibrillation is assumed to be the mean circulatory pressure [23, 24]. It seems that the pressures converge after 7 s [24], even though longer periods have been reported, perhaps because of flow limitation [23]. More recently, Pmsf was measured in intensive care units on cadavers one minute after an expected death [29, 32]. Note that RVr is not estimated by this method (see Additional file 1).

### The heart–lung interactions method

**Fig. 3:**
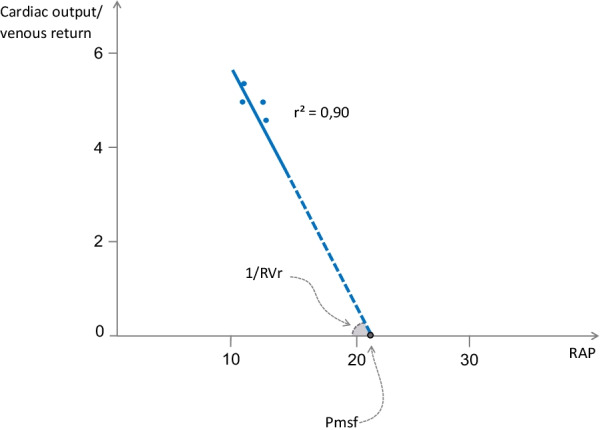
Example of a venous return curve obtained by the heart–lung interactions method. In this example, end-expiratory and end-inspiratory holds were performed. The 4 pairs of values of central venous pressure and cardiac output obtained during these holds were plotted on a graph, with cardiac output on the y-axis and central venous pressure on the x-axis. The regression line of these points could be equated to the venous return curve. The mean systemic filling pressure (Pmsf) was estimated as the pressure corresponding to the x-intercept of the extrapolated regression line. The resistance to venous return (RVr) was estimated as the inverse of the slope of the regression line

This method was developed in intubated patients by Maas and colleagues, who cleverly took advantage of heart–lung interactions [33, 34]. During an end-inspiratory hold, the intrathoracic pressure increases RAP (estimated through the CVP) and decreases cardiac output (a surrogate of venous return). In contrast, during an end-expiratory hold, RAP decreases and cardiac output increases (Fig. 3). Then, the pairs of CVP and cardiac output values are plotted on a graph (see Additional file 1). The regression line between these points estimates the venous return curve. Pmsf is estimated by the CVP value at intersection of the x-axis, and RVr is estimated as the inverse of the slope [29].

This method, of course, has limitations, even though it provides a qualitative assessment of the venous return curve determinants. It might be affected by the time delay between the arterial pulse pressure measurement and the venous return flow. Any error in the measurement of CVP may also introduce a large error in the estimation of the curve slope and in Pmsf. The method has also been suspected to overestimate Pmsf, and its accuracy may be influenced by the volume status [20].

### The transient stop-flow arm method

This method reproduces the cardiac arrest method at the arm level. After a rapid occlusion through an arm cuff, the arterial and venous pressures measured downstream equilibrate at a pressure level which estimates Pmsf. This method has been validated in humans compared to the heart–lung interactions method [35]. However, it might be limited by the fact that the venous compartment is less compliant in the muscle than in the splanchnic vascular bed [16], and the method may also be considered as more qualitative than quantitative.

## Mathematical estimation: Parkin and Leaning’s method

An analogue of Pmsf can be estimated from the real values of mean arterial pressure, RAP and cardiac output using the formula: Pmsf_(analogue)_ = *a* x RAP + *b* x mean arterial pressure + *c* x cardiac output. In this formula, *a* and *b* are dimensionless constants (*a* + *b* = 1) [36]. The variations of Pmsf estimated by this method during volume expansion are consistent with Guyton’s model, suggesting its validity [35, 37]. The fact that it does not assess RVr may affect its reliability. However, the method has also been validated against Pmsf determined at full arterio-venous pressure equilibrium [38] and against Pmsf estimated by the heart–lung interactions method [39].

## What are the typical patterns of venous return determinants during common clinical situations?

### Hypovolaemic shock

**Fig. 4:**
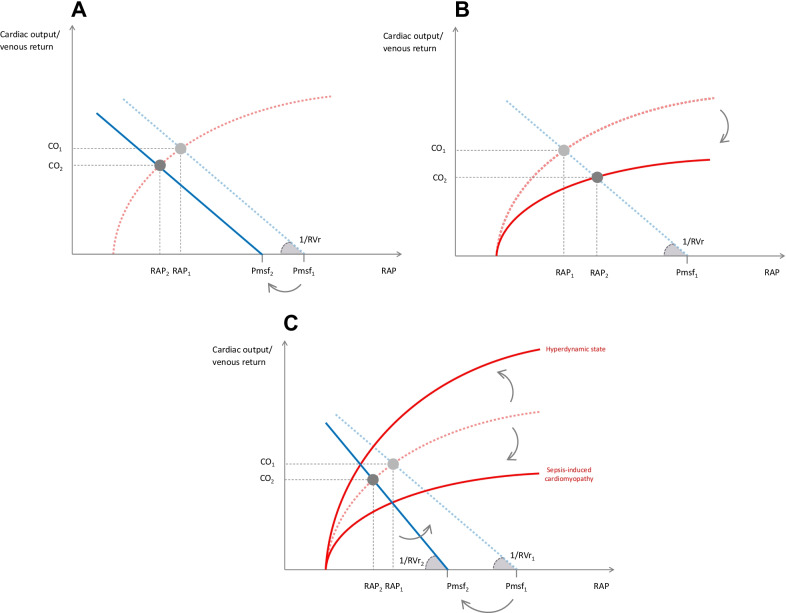
Effects on venous return and its determinants of typical clinical situations. **A** Hypovolaemic shock. Hypovolaemia decreases the stressed volume and therefore the mean systemic filling pressure (Pmsf). Then, the gradient between Pmsf and right atrial pressure (RAP) decreases, while the resistance to venous return (RVr) is not modified. As a consequence, the equilibrium point is shifted to the bottom left, and cardiac output (CO) and venous return decrease. **B** Cardiogenic shock. The slope of the Frank–Starling curve decreases. The equilibrium point is shifted to the bottom right, following the new, flattened, Frank–Starling curve. Right atrial pressure (RAP) increases, whereas mean systemic filling pressure (Pmsf) and resistance to venous return (RVr) are not modified. The (Pmsf–RAP) gradient decreases, reducing venous return and cardiac output (CO). **C** Septic shock. Vasodilation increases the venous capacitance, which decreases mean systemic filling pressure (Pmsf). Consequently, the gradient between Pmsf and right atrial pressure (RAP) decreases. Resistance to venous return (RVr) may also decrease. As a consequence, venous return and cardiac output (CO) decrease. A “hyperdynamic state”, with increased cardiac contractility, may steepen the Frank–Starling curve, while septic myocardial dysfunction may flatten it. Dashed lines and the index “1” indicate the normal state. Solid lines and the index “2” correspond to the pathological state

During hypovolaemia, the stressed blood volume decreases along with the total blood volume. Pmsf decreases whereas RVr is not modified [33] (Fig. 4A). The equilibrium point moves to the bottom left, and the line shifts to the left without changing its slope. In the case of preload responsiveness, venous return and cardiac output decrease. In the case of hypotension, this phenomenon is quickly counteracted by the sympathetic stimulation, which recruits the physiological reserve of unstressed blood volume, acting like a “self-volume expansion”.

### Cardiogenic shock

Systolic ventricular dysfunction decreases the slope of the Frank–Starling curve (Fig. 4B) without changing Pmsf and RVr. The point of equilibrium shifts to the bottom right, following the new Frank–Starling curve: RAP increases, and the (Pmsf–RAP) gradient decreases (Fig. 4B)[3].

Once sympathetic reflex stimulation occurs, two main effects appear. First, the venous capacitance decreases, and therefore, Pmsf increases. However, this mechanism has little effect. Indeed, the equilibrium point is on the flat part of the Frank–Starling curve, so that RAP increases to a similar extent as Pmsf and the venous return gradient does not increase significantly. For the same reason, volume expansion theoretically cannot restore cardiac output in this situation. The second mechanism is the sympathetic recruitment of a contractility reserve, which tends to increase the slope of the Frank–Starling curve.

Dobutamine affects the three determinants of venous return. It increases the slope of the cardiac function curve. Along with the decrease in right ventricular afterload by pulmonary vasodilation [40], this decreases RAP [41]. Secondly, dobutamine induces venous vasodilation through the vascular beta-adrenergic receptors [42–44]. Nevertheless, these effects are overwhelmed by the inotropic effect, so that cardiac output increases [41, 42, 44, 45].

### Septic shock

Strong venous dilation increases the unstressed blood volume and decreases Pmsf. The capillary leak contributes to this phenomenon (Fig. 4C) [46, 47]. Animal studies have found moderate and non-significant decreases in RVr [28] [46], but the models of circulatory failure were not hyperdynamic. In humans with septic shock and increased cardiac output, vasodilation may be responsible for a strong decrease in RVr.

Cardiac function may also be affected. Arterial vasodilation decreases the left ventricular afterload, thereby increasing the slope of the Frank–Starling curve. This may explain an increase in cardiac output during the classic “hyperdynamic state” (Fig. 4C). This increase in cardiac output may also be partly linked to the decrease in RVr. In contrast, sepsis-induced cardiac systolic dysfunction [48] flattens the Frank–Starling curve, tending to decrease venous return and cardiac output in association with the decrease in Pmsf (Fig. 4C).

### Mechanical ventilation

The effects of ventilation on venous return are probably different depending on whether one considers tidal ventilation or the effect of high intrathoracic pressures [25]. An increase in positive end-expiratory pressure (PEEP), transmitted to the right atrium, increases RAP. This is accompanied by an increase in Pmsf, due to the transmission of pressure to the splanchnic pressure. This increase in Pmsf does not completely compensate for the concomitant increase in RAP, but the difference is negligible, so that venous return does not change significantly [30].

An increase in RVr with high levels of PEEP has been described [30, 49]. It is believed to result from flow limitation, which collapses the large veins at their junction with the right atrium. However, this increase in RVr has only been reported in studies where high PEEP levels have been used [25, 30]. It seems to be absent at PEEP levels ≤ 10 cmH_2_O [30]. Thus, the decrease in cardiac output induced by moderate PEEP levels is primarily due to an increase in right ventricular afterload.

The effects of tidal ventilation on Pmsf appear to be minimal. However, small variations have been described [31] possibly due to a blood volume transfer from the pulmonary to the venous compartment [25].

## How can the physiology of venous return change practice?

### Volume expansion and the importance of central venous pressure

As has been shown in critically ill patients, fluid administration has the exact opposite effect of hypovolaemia, as it increases stressed blood volume and Pmsf [50]. Contrary to what one might expect from the decrease in sympathetic tone, RVr remains unchanged [33, 51, 52].

**Fig. 5:**
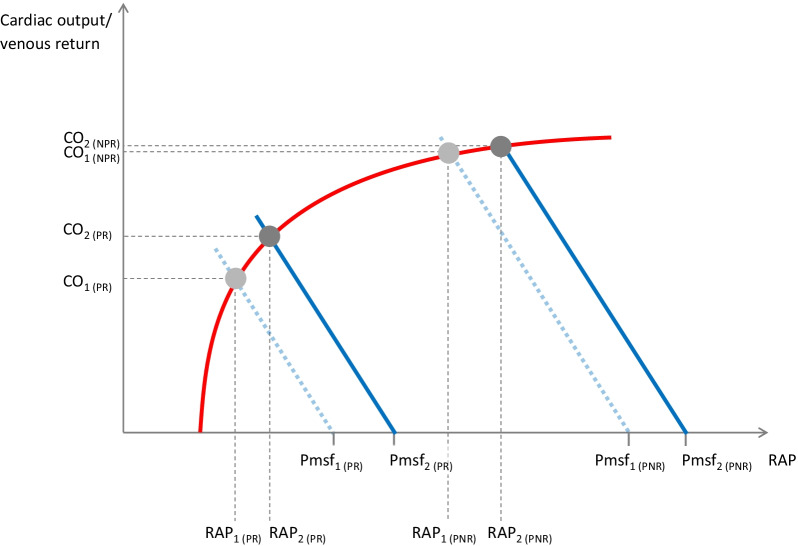
Effects of volume expansion on venous return and its determinants depending on the degree of preload responsiveness. Volume expansion increases the stressed blood volume and, then, increases mean systemic filling pressure (Pmsf). The equilibrium point is shifted to the upper right. **A** In the case of preload responsiveness (PR), Pmsf increases to a larger extent than right atrial pressure (RAP), inducing an increase in the (Pmsf–RAP) gradient. Venous return and cardiac output (CO) increase. **B** In the case of preload non-responsiveness (PNR), RAP increases to a similar extent as Pmsf, the (Pmsf–RAP) gradient remains unchanged and CO does not increase significantly

However, patients with and without preload responsiveness behave differently. Our group has shown that a fluid bolus increases Pmsf, to a similar extent whatever the degree of preload responsiveness [53]. In the case of preload responsiveness, as the Frank–Starling curve is steep, volume expansion increases Pmsf more than it increases RAP (Fig. 5). The (Pmsf–RAP) gradient increases. As RVr is unchanged, venous return and cardiac output increase. In the case of preload unresponsiveness, volume expansion increases Pmsf to the same extent. However, the increase in cardiac preload is not transformed into an increase in stroke volume. The end-diastolic ventricular pressure increases, leading to a substantial increase in RAP. Then, RAP and Pmsf increase to a similar extent and the gradient of venous return remains unchanged (Fig. 5). These results were confirmed by three other studies using methods other than heart–lung interactions [37, 54, 55].

Understanding these physiological effects of fluid helps to interpret CVP during fluid infusion. It is often thought that CVP should increase during fluid administration. However, we have seen that on the contrary, in preload responders, CVP should not increase so much, in order to allow the venous return gradient to increase [53]. The same mistake has been made regarding passive leg raising (PLR). It is physiologically wrong that PLR is not efficient enough if CVP does not increase [56], as has been suggested [57].

### Passive leg raising is really a “pseudo fluid challenge”

PLR mimics a fluid challenge by transferring some venous blood from the lower limbs and the splanchnic vascular bed towards the heart [58]. Two clinical studies have shown that Pmsf increases during PLR without changing RVr [52, 53], by increasing the stressed blood volume, as does volume expansion. The determinants of venous return behave during PLR as they do during fluid infusion, depending on the degree of preload responsiveness [53].

It has been shown that PLR may suffer from false negatives in patients with intra-abdominal hypertension [59]. This can be explained in light of the effects of PLR on venous return: intra-abdominal hypertension decreases the volume of the splanchnic vascular bed and limits the recruitment of stressed blood volume by PLR. In addition, in some patients with very high intra-abdominal hypertension and deep hypovolaemia, venous return might be limited by compression of the inferior vena cava, creating a flow limitation phenomenon [60].

### Revisited effects of norepinephrine

Norepinephrine is usually administered with the aim of increasing arterial tone and mean arterial pressure. Nonetheless, data suggest that its venous effects are also significant [28, 61–63]. The recruitment of unstressed blood volume may, in theory, be equivalent to a volume expansion of one litre [12]. Clinical studies have shown that norepinephrine actually increases cardiac preload, resulting in an increase in cardiac output in patients with preload responsiveness [64, 65].

This increase in cardiac preload is due to the recruitment of unstressed volume through venoconstriction, which reduces the venous capacitance [20]. It should be noted that this effect is short-lived due to capillary leak. A recent study has shown that the Pmsf already begins to decline 40 min after an initial increase induced by norepinephrine [20]. Finally, through β1 stimulation, norepinephrine also exerts an inotropic effect which may contribute to the increase in cardiac output [66]. However, these effects might be counteracted by a norepinephrine-induced increase in RVr.

Animal studies suggest that norepinephrine increases Pmsf [28, 67, 68]. In an endotoxin model of septic shock in pigs, Datta and Magder reported that this increase in Pmsf was not accompanied by any increase in RVr [28]. Similarly in septic patients, our group demonstrated that decreasing the dose of norepinephrine decreased Pmsf and decreased RVr to a lesser extent. As a consequence, the (Pmsf–RAP) gradient decreased, inducing a decrease in venous return and cardiac output in preload-responsive patients [61].

In contrast, increasing the dose of norepinephrine in septic shock patients should increase Pmsf while modestly increasing RVr. This smaller effect of norepinephrine on RVr than on Pmsf has been attributed to the fact that the activation of α-adrenergic receptors constricts the venous drainage of the splanchnic vascular bed [69]. Although the effects of epinephrine might be similar to those of norepinephrine, at least in animals [70], phenylephrine, which is a pure alpha-agonist, likely exerts different effects [69].

Interestingly, different effects of norepinephrine were described in another setting. In post-operative cardiac surgery patients, increasing the dose of norepinephrine decreased cardiac output in half of the patients because the RVr increase was larger than the Pmsf increase [63]. This different effect of norepinephrine in cardiac surgery patients might be explained by a different degree of vasodilation compared to septic shock.

Analysis of the effects of the volume expansion in light of the physiology of venous return helps us understand the synergy that exists between volume expansion and norepinephrine. Both norepinephrine and fluid contribute to the increase in Pmsf. More than this “additive” effect, norepinephrine and fluid infusion may act synergistically. Once norepinephrine has been started or increased, any additional fluid infusion may increase Pmsf to a larger extent than before. The fluid-induced increase in stressed blood volume occurs when fluid is administered in a constricted venous network rather than in a large, dilated venous tank. This is what is suggested by a study in septic shock patients, showing that PLR increases Pmsf to a larger extent at a higher than at a lower dose of norepinephrine [62].

All these arguments advocate early administration of norepinephrine, along with fluids, during septic shock resuscitation, especially in patients with severe hypotension. Not only is norepinephrine the only means of restoring arterial pressure rapidly, but also it potentiates the effects of fluid administration. This may lead to reduction in the total amount of fluid administered for resuscitation and thereby might improve outcome [71].

### Prone positioning

Though prone positioning has become a cornerstone of the management of acute respiratory distress syndrome, its haemodynamic effects have scarcely been described. First, prone positioning reduces pulmonary arterial resistance and reduces right ventricular afterload [72, 73]. This likely results from different factors, such as a reduction in hypoxic and hypercapnic vasoconstriction and the filling of the pulmonary vessels, which increases the proportion of vessels in non-1 zones of West [74]. Second, prone positioning also increases cardiac preload, through the increase in intra-abdominal pressure, and thus cardiac output in patients with fluid responsiveness [73, 75]. This effect is not negligible, as cardiac output might increase by 20% in case of preload responsiveness [73], in the same range as a 500-mL saline infusion [53].

Our group has shown that Pmsf increased in all patients with acute respiratory distress syndrome during prone positioning [76]. In the case of fluid responsiveness, Pmsf increased more than RAP, allowing the (Pmsf–RAP) gradient to increase. For most patients, the increase in RVr following the increase in intra-abdominal pressure was moderate, much lower than the increase in the (Pmsf–RAP) gradient. Therefore, venous return and cardiac output increased in preload-responsive patients. In the case of no preload responsiveness, RAP increased to a similar extent as Pmsf, so that the (Pmsf–RAP) gradient, venous return and cardiac output remained unchanged [76]. However, in a few preload-responsive patients, the increase in RVr was so large that it overwhelmed the increase in the (Pmsf–RAP) gradient [76]. For these few patients, even in the case of preload responsiveness, venous return and cardiac output did not increase with prone positioning [76]. In fact, the increase in RVr likely depended on the increase in intra-abdominal pressure, but also on the central blood volume, since hypovolaemia may promote flow limitation through the occlusion of the inferior vena cava during prone positioning.

## Conclusion

Venous return plays a major role in cardiovascular physiology, and its determinants, Pmsf, RAP and RVr, are major haemodynamic variables. Guyton's model helps describe their interaction. While the determinants of venous return were for a long time examined only in animal studies, innovative methods have recently been developed to estimate them in critically ill patients. These methods have helped us understand the value of CVP in the assessment of the response to fluid. Also, the venous effect of norepinephrine has been clearly demonstrated during septic shock, thus advocating its administration along with fluids in hypotensive patients. Finally, venous return physiology explains the significant increase in cardiac preload during prone positioning.

## Supplementary Information

**Additional file 1.** How to measure the determinants of venous return? Detailed methods.

## Data Availability

Not applicable.

## Abbreviations

CO: Cardiac output
CVP: Central venous pressure
PEEP: Positive end-expiratory pressure
PLR: Passive leg raising
Pmsf: Mean systemic filling pressure
RAP: Right atrial pressure
RVr: Resistance to venous return
VR: Venous return
