# Supplementary material for: Venous return and mean systemic filling pressure: physiology and clinical applications
Source: Crit Care. 2022 May 24;26:150. doi: 10.1186/s13054-022-04024-x (PMC9128096; doi:10.1186/s13054-022-04024-x)
Supplement: Supplementary file 1 — Additional file 1. How to measure the determinants of venous return? Detailed methods. [file 13054_2022_4024_MOESM1_ESM.docx]

Venous return and mean systemic filling pressure: physiology and clinical applications.

Romain PERSICHINI^1^, Christopher LAI^2^, Jean-Louis TEBOUL^2^, Imane ADDA^2^, Laurent GUÉRIN^2^, and Xavier MONNET^2^

1 : Service de Réanimation et Soins Continus, Centre Hospitalier de Saintonge, Saintes, France.

2 : Université Paris-Saclay, AP-HP, Service de médecine intensive-réanimation, Hôpital Bicêtre, DMU CORREVE, Inserm UMR S_999, FHU SEPSIS, Groupe de recherche clinique CARMAS, Le Kremlin-Bicêtre, France

Additional file 1

# How to measure the determinants of venous return?

For years, estimation of mean systemic filling pressure (Pmsf) and resistance to venous return (RVr) was not possible in the critically ill, though it is in these patients that the clinical relevance of the physiology of venous return is the most important. In recent years, innovative methods have been developed to approach the determinants of venous return at the bedside. They are of no interest for the routine management of patients, but their use in physiological studies has made it possible to better describe the effects of some common therapeutic interventions.

## Guyton’s animal model

Guyton’s seminal studies were performed in anaesthetised dogs. The heart was replaced by an extracorporeal circulatory bypass. A pump located outside the chest allowed the variation of right arterial pressure (RAP). A temporary stop of the pump allows the measurement of the mean circulatory filling pressure. The venous return curve was obtained by varying the right atrial pressure (RAP) and the mean systemic filling pressure (Pmsf). The level of RAP was varied by changing the minute capacity of the pump. the mean circulatory filling pressure was varied by increasing or decreasing the total quantity of blood in the circulatory system. The venous resistance was applied by progressive occlusion of the veins entering the right atrium [1,2].

Other animal studies constructed venous return curves varying the RAP with an inflatable balloon in the right atrium [3] or by increasing the intrathoracic pressure [4]. Then, cardiac output was measured, and venous return curves were constructed assuming that both were equal at equilibrium. Other animal studies measured Pmsf during cardiac arrest induced by ventricular fibrillation [5]. Finally, the mean circulatory filling pressure was measured in the cardiovascular system of animals after injection of acetylcholine boluses inducing asystole [5].

## Cardiac arrest

In humans, the first measurements of Pmsf were made during electrophysiologic diagnostic investigations. Arterial pressure and central venous pressure (CVP) following a cardiac arrest induced by ventricular fibrillation were recorded and assumed to be equal to Pmsf [6,7]. It seems that the pressures converge after 7 sec [7], even though longer periods have been reported, perhaps because of flow limitation [6]. This method is of course not suitable at the bedside for patients with shock. Moreover, provided that cardiac output is not measured, RVr is not estimated.

More recently, Pmsf was measured in intensive care units on cadavers one minute after an expected death in a context of care withholding [8,9]. However, the value of Pmsf found with this method, recorded while the vasomotor tone of these patients was severely altered, and while the ventilator was disconnected in one study [9], cannot be extrapolated to living patients.

## The heart-lung interactions method

Maas and colleagues have developed a method which estimates the venous return curve by using the changes in cardiac loading conditions induced by mechanical ventilation in intubated patients [10,11].

During an end-inspiratory hold, the intrathoracic pressure increases RAP and decreases cardiac output. In contrast, during an end-expiratory hold, RAP decreases and cardiac output increases. RAP is estimated through the CVP, and cardiac output is considered as a surrogate of venous return (Figure 3). Then, the pairs of values of CVP and cardiac output obtained during these successive respiratory holds are plotted on a graph, with CVP values on the y-axis and cardiac output values on the x-axis. Finally, the regression line between these points is drawn and is considered an estimation of the steep part of the venous return curve. This line is extrapolated to the x-axis, and Pmsf is estimated by the CVP value at intersection of both. RVr is estimated as the inverse of the slope of the regression line. For trained teams, the measurement of CVP and cardiac output during the 4 inspiratory and expiratory holds takes less than 10 minutes, so that the equilibrium state is still present during this short period.

The use of high levels of airway pressure may prevent the use of the technique at the bedside. Moreover, they may induce large decreases in cardiac output. Nevertheless, our group modified this method by performing respiratory holds at two levels of positive end-expiratory pressure (PEEP), a low PEEP level of 5 cmH_2_O and a high PEEP level titrated to obtain a maximum of 30 cmH_2_O. As a consequence, the plateau pressure is always under 30 cmH_2_O and the range of CVP and cardiac output changes in enlarged [12–14].

Some authors have argued that variation of intrathoracic pressure could modify Pmsf by activation of the baroreflex [15]. However, the heavy sedation used in these patients decreases this effect. It should also be noted that this method may overestimate the value of Pmsf compared to the balloon occlusion method in animals [16]. Another limitation of this method is that its accuracy could be affected by the underlying volume state. The best concordance is obtained with the euvolaemic state and the worst with the hypervolaemic state [17]. Nevertheless, the heart-lung interactions method tracks changes of Pmsf concordantly compared to the balloon occlusion method [17]. Lastly, this method may be considered as a qualitative approach more than as a purely quantitative one. Indeed, difficulties in measuring CVP precisely could lead to mistakes when plotting points on the VR curve. The prerequisite of this method is therefore to ensure a very precise measurement of cardiac output, but also above all of CVP [18].

To conclude, the heart-lung interactions method is the only one which allows assessment at the same time of Pmsf and RVr at the bedside. It is the most widely used method to date and could be considered as a reference for critically ill patients [8].

## The transient stop-flow arm arterial-venous equilibrium

This method reproduces the cardiac arrest method at the arm level. After a rapid occlusion through a pneumatic cuff, the arterial pressure measured through a radial catheter, and the venous pressure measured through a catheter in a peripheral vein, equilibrate at a pressure level which estimates Pmsf.

This method has been validated in humans compared to heart-lung interactions method [19] and recently in animals compared to the reference zero-flow method [20]. Considering the difficulties in measuring pressure variations precisely, as for the heart-lung interactions method, the transient stop-flow arm method is more qualitative than quantitative.

The main interest of this method is that it can be performed in awake patients with spontaneous breathing. Its main limitation is that it measures only Pmsf, without any estimation of RVr.

## Mathematical estimation of Pmsf

Pmsf can be estimated from the real values of mean arterial pressure, RAP, cardiac output and anthropometric data (weight, height, age) by means of a formula established using a mathematical approach based on the Guytonian model [21]:

Pmsf (analogue) = *a* x mean arterial pressure + *b* x RAP + *c* x cardiac output.

In this formula, *a* and *b* are dimensionless constants (with *a* + *b* = 1), whereas *c* is a constant of resistance based on anthropometric data. A virtual stopping of cardiac output in the equation allows the estimation of the Pmsf analogue. The equation was integrated in a specific device, which is no longer marketed.

The main limitation of this method is that resistance variables are estimated from anthropometric data only, so that it may not be suitable for critically ill and unstable patients. Nevertheless, the variations of Pmsf estimated by this method during volume expansion were consistent with Guyton’s model, suggesting it is valid [19,22]. However, RVr is not estimated by this method.

## References

1. Guyton AC. Determination of cardiac output by equating venous return curves with cardiac response curves. Physiol Rev. 1955;35:123–9.

2. Guyton AC, Lindsey AW, Abernathy B, Richardson T. Venous return at various right atrial pressures and the normal venous return curve. Am J Physiol. 1957;189:609–15.

3. Datta P, Magder S. Hemodynamic response to norepinephrine with and without inhibition of nitric oxide synthase in porcine endotoxemia. Am J Respir Crit Care Med. 1999;160:1987–93.

4. Fessler HE, Brower RG, Wise RA, Permutt S. Effects of positive end-expiratory pressure on the canine venous return curve. Am Rev Respir Dis. 1992;146:4–10.

5. Lee RW, Lancaster LD, Gay RG, Paquin M, Goldman S. Use of acetylcholine to measure total vascular pressure-volume relationship in dogs. Am J Physiol. 1988;254:H115-119.

6. Schipke JD, Heusch G, Sanii AP, Gams E, Winter J. Static filling pressure in patients during induced ventricular fibrillation. Am J Physiol Heart Circ Physiol. 2003;285:H2510-2515.

7. Kottenberg-Assenmacher E, Aleksic I, Eckholt M, Lehmann N, Peters J. Critical closing pressure as the arterial downstream pressure with the heart beating and during circulatory arrest. Anesthesiology. 2009;110:370–9.

8. Wijnberge M, Sindhunata DP, Pinsky MR, Vlaar AP, Ouweneel E, Jansen JR, et al. Estimating mean circulatory filling pressure in clinical practice: a systematic review comparing three bedside methods in the critically ill. Ann Intensive Care. 2018;8:73.

9. Repessé X, Charron C, Fink J, Beauchet A, Deleu F, Slama M, et al. Value and determinants of the mean systemic filling pressure in critically ill patients. Am J Physiol Heart Circ Physiol. 2015;309:H1003-1007.

10. Maas JJ, Geerts BF, van den Berg PCM, Pinsky MR, Jansen JRC. Assessment of venous return curve and mean systemic filling pressure in postoperative cardiac surgery patients. Crit Care Med. 2009;37:912–8.

11. Jansen JRC, Maas JJ, Pinsky MR. Bedside assessment of mean systemic filling pressure. Curr Opin Crit Care. 2010;16:231–6.

12. Persichini R, Silva S, Teboul J-L, Jozwiak M, Chemla D, Richard C, et al. Effects of norepinephrine on mean systemic pressure and venous return in human septic shock. Crit Care Med. 2012;40:3146–53.

13. Guérin L, Teboul J-L, Persichini R, Dres M, Richard C, Monnet X. Effects of passive leg raising and volume expansion on mean systemic pressure and venous return in shock in humans. Crit Care. 2015;19:411.

14. Lai C, Adda I, Teboul J-L, Persichini R, Gavelli F, Guérin L, et al. Effects of Prone Positioning on Venous Return in Patients With Acute Respiratory Distress Syndrome. Crit Care Med. 2021;49:781–9.

15. Repessé X, Charron C, Geri G, Aubry A, Paternot A, Maizel J, et al. Impact of positive pressure ventilation on mean systemic filling pressure in critically ill patients after death. J Appl Physiol. 2017;122:1373–8.

16. Berger D, Moller PW, Weber A, Bloch A, Bloechlinger S, Haenggi M, et al. Effect of PEEP, blood volume, and inspiratory hold maneuvers on venous return. Am J Physiol Heart Circ Physiol. 2016;311:H794-806.

17. Werner-Moller P, Sondergaard S, Jakob SM, Takala J, Berger D. Effect of volume status on the estimation of mean systemic filling pressure. J Appl Physiol. 2019;126:1503–13.

18. Magder S. Central venous pressure monitoring. Curr Opin Crit Care. 2006;12:219–27.

19. Maas JJ, Pinsky MR, Geerts BF, de Wilde RB, Jansen JR. Estimation of mean systemic filling pressure in postoperative cardiac surgery patients with three methods. Intensive Care Med. 2012;38:1452–60.

20. Werner-Moller P, Heinisch PP, Hana A, Bachmann KF, Sondergaard S, Jakob SM, et al. Experimental validation of a mean systemic pressure analog against zero-flow measurements in porcine VA-ECMO. J Appl Physiol. 2022;132:726–36.

21. Parkin WG, Leaning MS. Therapeutic control of the circulation. J Clin Monit Comput. 2008;22:391–400.

22. Cecconi M, Aya HD, Geisen M, Ebm C, Fletcher N, Grounds RM, et al. Changes in the mean systemic filling pressure during a fluid challenge in postsurgical intensive care patients. Intensive Care Med. 2013;39:1299–305.
